# When an Extra X Means Extra Risk: Venous Thromboembolism in Klinefelter Syndrome

**DOI:** 10.7759/cureus.115880

**Published:** 2026-09-07

**Authors:** Rama AlMasri, Ankit Kulkarni, Niket Shah, Nadeen Saad, Jason P Law, Waleed Shaker, Kelsey MacDonald, Heather Laird Fick

**Affiliations:** 1 Internal Medicine, Michigan State University, East Lansing, USA

**Keywords:** hypercoagulability, klinefelter syndrome, pulmonary embolism, sex chromosome aneuploidy, testosterone replacement therapy, thrombophilia, venous thromboembolism

## Abstract

Klinefelter syndrome (KS) is a common but frequently underdiagnosed sex chromosome aneuploidy that confers a markedly elevated risk of venous thromboembolism (VTE), yet it remains absent from major anticoagulation guidelines. We present a 41-year-old man with known KS on testosterone replacement who developed massive bilateral pulmonary embolism with concurrent deep vein thrombosis, presenting with syncope, dyspnea, and right heart strain on imaging. Hematocrit was 48.8% at admission, below the erythrocytosis threshold despite ongoing testosterone therapy. Serial laboratory data over 13 months consistently showed normal hematocrit values, implicating KS-intrinsic hypercoagulability rather than testosterone-induced erythrocytosis as the primary thrombotic driver. Thrombophilia testing was negative for factor V Leiden, prothrombin G20210A, and JAK2 mutations. The patient was managed with heparin bridged to apixaban and discharged on lifelong anticoagulation. This case highlights KS as a high-risk thrombophilic condition in which the underlying genetic syndrome, not testosterone therapy, is the primary driver of thrombosis. Early recognition and formal inclusion of KS in VTE management guidelines are needed to standardize care for this commonly underdiagnosed population.

## Introduction

Klinefelter syndrome (KS) is a sex chromosome aneuploidy caused by the presence of one or more supernumerary X chromosomes, most commonly resulting in a 47,XXY karyotype, and is the most common chromosomal disorder in males [1]. It affects approximately 1 in 500 to 1,000 individuals, yet up to 75% of affected individuals remain undiagnosed throughout their lifetime [1]. The clinical phenotype is broad and includes hypogonadism, infertility, gynecomastia, metabolic syndrome, and an increased risk of autoimmune and cardiovascular diseases [1]. Despite this, KS is most often recognized for its reproductive consequences, while its systemic complications remain underappreciated [2].

KS affects roughly 1 in 500-1,000 males [1], but the thrombotic risk remains underrecognized. In a nationwide Swedish study, men with KS were about 12 times more likely to develop venous thromboembolism (VTE) before the age of 30, and by age 70, 20.8% had experienced a clot versus 2.8% of controls [2]. A 2023 biobank study demonstrated a fourfold to fivefold increase in VTE risk associated with supernumerary sex chromosome aneuploidies [3]. This risk exceeds that associated with many established thrombophilias, yet KS does not appear in anticoagulation guidelines. The hypercoagulable state in KS is thought to arise from a combination of X-chromosome gene dosage effects on coagulation factor expression, testosterone deficiency-related endothelial dysfunction, and metabolic comorbidities, including obesity and insulin resistance [1,4]. Paradoxically, patients with KS show largely normal thrombin generation despite their extreme clinical risk [5]. This case illustrates this paradox and supports lifelong anticoagulation in KS following a first VTE event.

## Case presentation

A 41-year-old Caucasian man with KS, previously confirmed by karyotype analysis (47,XXY), presented after a syncopal episode while climbing stairs. His medical history was significant for type 2 diabetes mellitus treated with metformin, hypertriglyceridemia, schizoaffective disorder treated with clozapine, a medication with well-documented prothrombotic risk, hypogonadism treated with weekly testosterone enanthate (75 mg intramuscularly), and a 10.5 pack-year smoking history.

He reported several weeks of progressively worsening left leg pain, exertional dyspnea, and chest pain prior to presentation. On examination, he was tachycardic with a pulse of 130 beats per minute; blood pressure was 137/71 mmHg, and oxygen saturation was 97% on room air.

Laboratory evaluation demonstrated an initial troponin level of 36 ng/L, which subsequently peaked at 774 ng/L. Hemoglobin was 16.7 g/dL with a hematocrit of 48.8%, without evidence of erythrocytosis, while the platelet count was 146 × 10³/µL. Serial laboratory values over the preceding 13 months are presented in Table 1.

**Table 1:** Serial laboratory values over the 13 months prior to and including the date of admission. Values marked “-” indicate that the test was not performed on that date. Reference range for adult male testosterone: 300-1,000 ng/dL. Therapeutic clozapine range: 350-600 ng/mL; values exceeding this range are highlighted in bold below.

Date	Testosterone (ng/dL)	Hematocrit (%)	Platelets (×10³/µL)	Clozapine (ng/mL)
4 weeks after admission	-	49.7	251	632
Admission day	-	48.8	146	-
3 weeks prior to admission	452	50.5	226	514
7 weeks prior to admission	-	50	254	649
4 months prior to admission	408	50.1	225	420
8 months prior to admission	577	49.8	250	582
12 months prior to admission	743	55.4	259	-
13 months prior to admission	1049	54.1	226	-

The differential diagnosis included pulmonary embolism, aortic dissection, chronic obstructive pulmonary disease (COPD) exacerbation, acute coronary syndrome, musculoskeletal pain, cardiac tamponade, and inherited thrombophilia. CT pulmonary angiography revealed extensive bilateral pulmonary emboli with a nonocclusive saddle embolus (Figure 1). Transthoracic echocardiography demonstrated severe right ventricular dilatation with reduced systolic function and McConnell's sign, consistent with acute right heart strain (Figure 2). Lower extremity venous USG confirmed a left lower extremity deep venous thrombosis.

**Figure 1:**
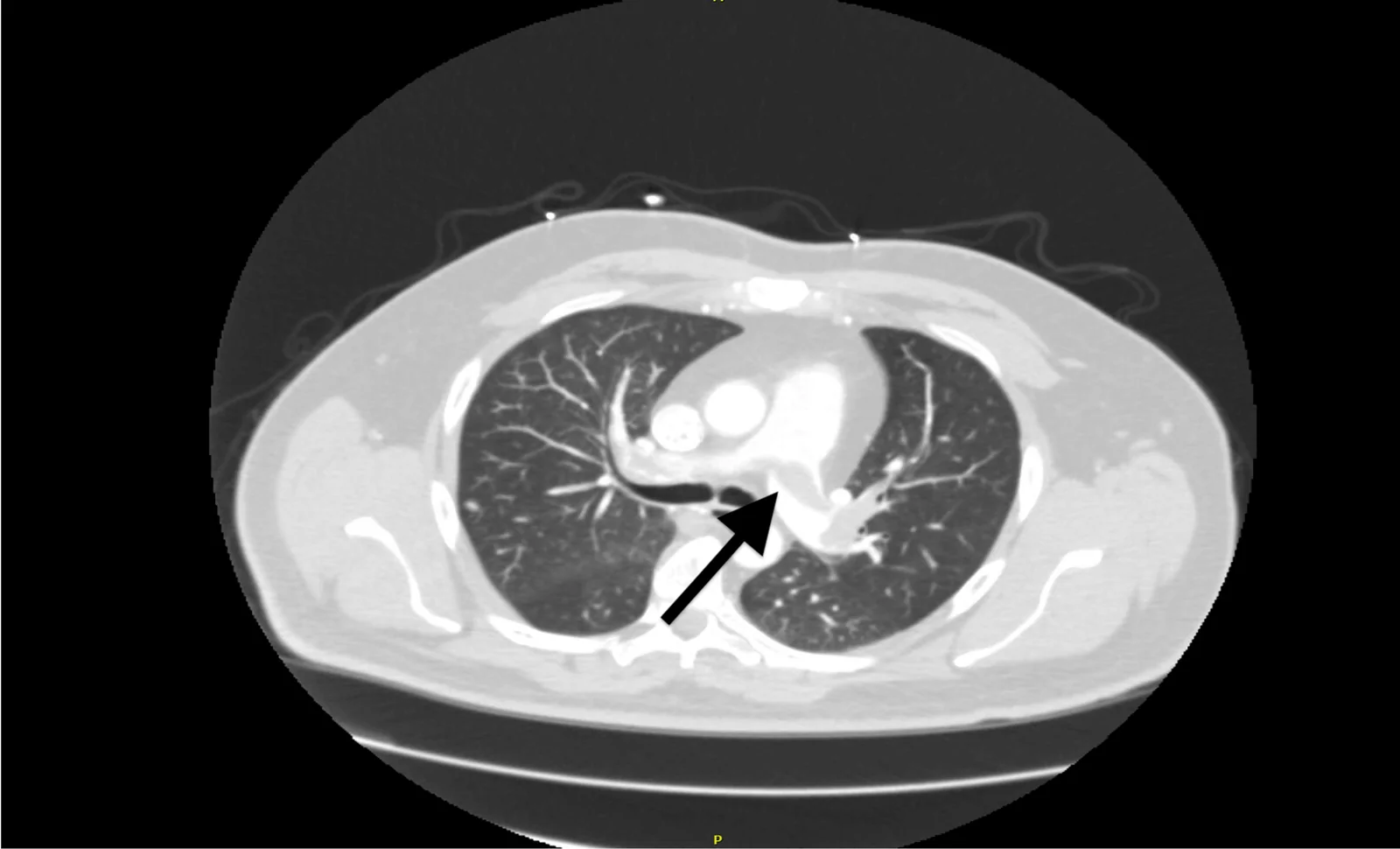
Axial CT pulmonary angiogram demonstrating extensive bilateral pulmonary emboli.

**Figure 2:**
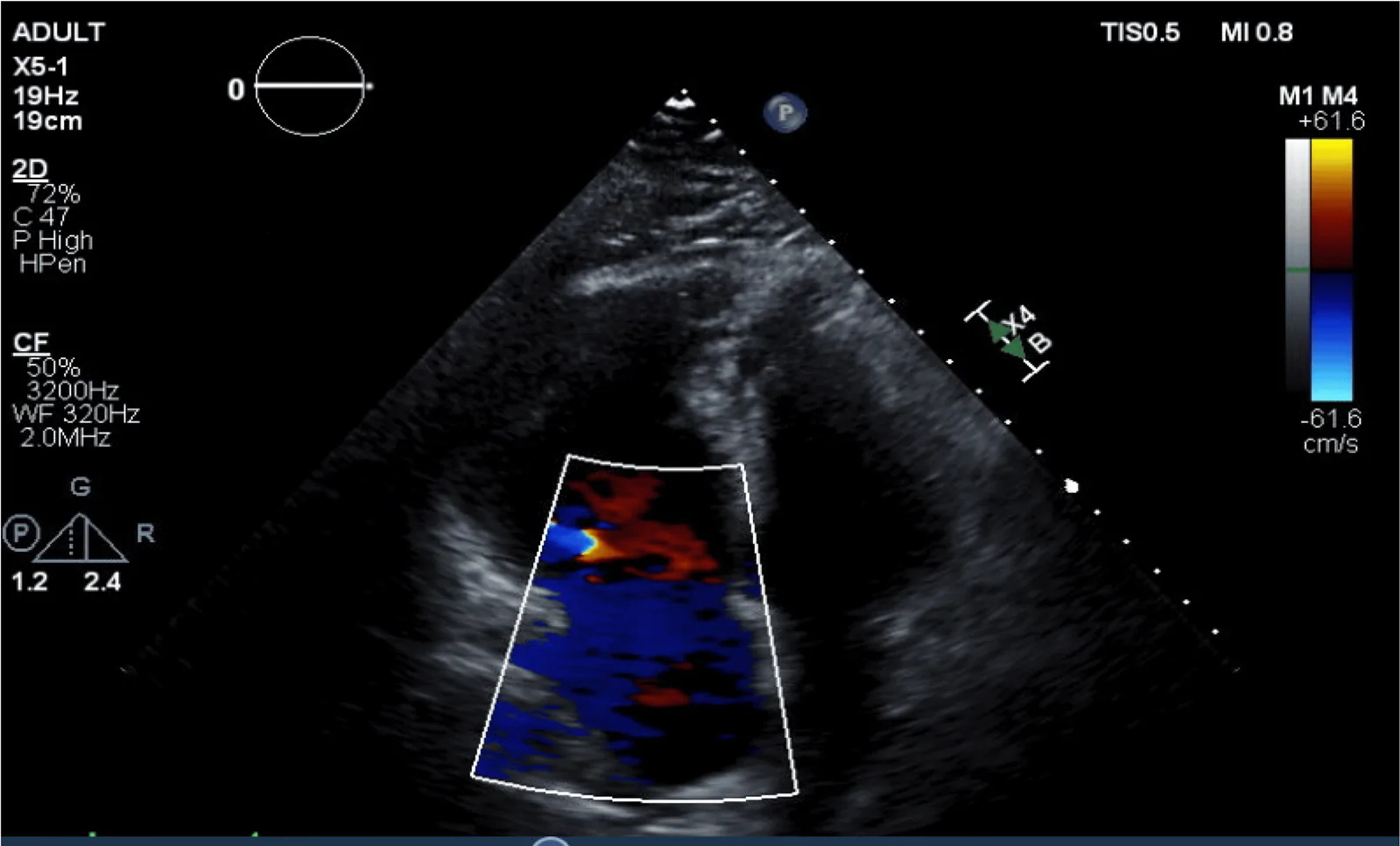
Severely dilated right ventricle (RV) with a positive McConnell sign.

The patient was classified as having intermediate-high-risk pulmonary embolism per European Society of Cardiology (ESC) guidelines, with a simplified Pulmonary Embolism Severity Index (sPESI) score of 1 (tachycardia ≥110 bpm), concurrent echocardiographic evidence of right ventricular dysfunction, and myocardial injury reflected by troponin elevation to 774 ng/L, collectively meeting criteria for systemic thrombolysis consideration. Although the patient met criteria for systemic thrombolysis, he declined tenecteplase after a thorough discussion of the associated bleeding risks. Interventional radiology was consulted; however, thrombectomy was not pursued given the extensive clot burden. The patient was managed with therapeutic IV heparin, transitioned to apixaban, and remained hemodynamically stable throughout hospitalization without requiring supplemental oxygen. He was discharged after four days in stable condition.

At the three-month follow-up, the patient remained clinically stable on apixaban 5 mg twice daily. A comprehensive thrombophilia panel returned negative for factor V Leiden, prothrombin G20210A mutation, and JAK2 mutation. Following endocrinology consultation, his testosterone dose was reduced from 75 mg to 50 mg weekly, with hematocrit remaining within normal limits on repeat testing. Given his underlying genetic predisposition and significant thromboembolic event, lifelong anticoagulation was recommended.

## Discussion

This patient's age falls within the range in which VTE is most frequently reported in KS, between 30 and 49 years [2]. The thrombotic risk in KS is multifactorial. The presence of an extra X chromosome may increase the expression of coagulation factors VIII and XI, both of which have been found to be elevated in patients with KS and VTE [2,4]. Plasminogen activator inhibitor-1 (PAI-1), which impairs fibrinolysis, may also be elevated [4], especially in the setting of obesity, insulin resistance, or hypogonadism. Some of these factors may have been relevant in this patient, who had type 2 diabetes [1]. His normal hematocrit at admission while receiving testosterone suggests that TRT-induced erythrocytosis was unlikely to have been the primary driver of the embolism.

KS is associated with an overall sixfold increase in VTE risk and up to a twelvefold increase in men younger than 30 years, with the standardized incidence ratio declining to 2.07 in those aged 70 years and older [2]. This age-dependent gradient is clinically important: the risk is highest during the decades when KS may remain undiagnosed or untreated, potentially compounding the risk for affected individuals.

The role of testosterone replacement therapy (TRT) in modulating this risk is bidirectional. Although TRT has been associated with erythrocytosis and alterations in coagulation dynamics, mechanistic data suggest a potentially protective effect. In a Danish cross-sectional study, testosterone-treated men had lower peak thrombin generation and lower endogenous thrombin potential than untreated men [5]. A national Danish cohort likewise observed fewer VTEs in testosterone-treated men, although the difference was not statistically significant [6]. Testosterone may reduce hypercoagulability by improving metabolic health and upregulating natural anticoagulants, including antithrombin, protein C, protein S, and tissue factor pathway inhibitor. Although one historical hematocrit value was 55.4% at 12 months before admission, subsequent values remained below the erythrocytosis threshold of 54%, including at admission. This finding argues against TRT-induced erythrocytosis as the primary driver at presentation.

A possible contributor in this case is clozapine, which carries a documented prothrombotic risk. Clozapine has been associated with an increased incidence of VTE. Proposed mechanisms include sedation-related immobility and stasis, metabolic adverse effects, and possible effects on platelet aggregation and fibrinolysis [7]. This patient's serum clozapine levels ranged from 420 to 649 ng/mL during the monitoring period, with values of 632 and 649 ng/mL intermittently exceeding the upper therapeutic reference threshold of 600 ng/mL. Therefore, clozapine may have represented an additional independent risk factor. The mild thrombocytopenia noted at admission (platelet count, 146 × 10³/µL, compared with prior values of 225-259 × 10³/µL) also warrants consideration. In the absence of prior heparin exposure, heparin-induced thrombocytopenia was unlikely. Possible explanations include consumptive thrombocytopenia associated with the acute massive pulmonary embolism or clozapine-related thrombocytopenia, a recognized but infrequent adverse effect. The mild degree of decline and its clinical context may support a consumptive etiology, although clozapine's contribution cannot be fully excluded.

The overall magnitude of VTE risk in KS invites comparison with established genetic thrombophilias. The standardized incidence ratio of 6.43 in KS [2] broadly overlaps with the three- to sevenfold increased risk reported for heterozygous factor V Leiden [8]. However, this comparison should be interpreted cautiously because these estimates may have been derived from different populations and measures of association. In younger patients, KS-associated VTE risk reaches twelvefold, exceeding the upper range typically reported for heterozygous factor V Leiden. In older patients, the KS-associated risk declines substantially, making the comparison less straightforward [2]. Nonetheless, unlike factor V Leiden, KS is not specifically mentioned in major anticoagulation or thrombophilia guidelines, despite being associated with a comparable or greater thrombotic burden during younger decades [8,9]. This disparity may represent a gap in evidence-to-practice translation.

The optimal duration of anticoagulation following VTE in KS has not been formally established by current guidelines. The ASH 2023 guidelines on thrombophilia testing recommend extended, and in many cases indefinite, anticoagulation in patients with high-risk thrombophilias, including antithrombin, protein C, and protein S deficiencies, where the annual VTE recurrence risk without anticoagulation is significantly elevated [9]. The chromosomal basis of KS constitutes a permanent, unmodifiable thrombotic risk factor that is conceptually analogous to these established indications, as it cannot be corrected through any intervention. Given the cumulative VTE incidence of 20.8% by age 70 in KS [2], the lifetime recurrence risk in this population plausibly meets or exceeds the threshold that triggers indefinite anticoagulation recommendations for other high-risk genetic thrombophilias [9]. On this basis, lifelong anticoagulation following a first VTE event in KS is a clinically evidence-informed recommendation, pending formal guideline recognition.

Despite these findings, only 48.7% of Danish men with KS had ever filled a testosterone prescription, and among those untreated, 72% were biochemically hypogonadal [6]. These data highlight the underrecognition of KS-related complications and support earlier diagnosis, consistent endocrine follow-up, and consideration of lifelong anticoagulation after a first thrombotic event, in alignment with recommendations for other genetic thrombophilias [9]. Clinicians managing patients with KS should maintain a low threshold for thrombophilia workup, monitor hematocrit and relevant medication levels, including clozapine where applicable, and engage hematology for anticoagulation planning following any thrombotic event.

## Conclusions

KS is a clinically significant but often overlooked chromosomal risk factor capable of causing life-threatening events such as massive pulmonary embolism, despite largely normal standard coagulation profiles. The permanent, unmodifiable nature of the 47,XXY karyotype creates a hypercoagulable state whose thrombotic burden is comparable in magnitude to that of well-established genetic thrombophilias, yet KS receives no formal recognition in major anticoagulation or thrombophilia guidelines. This case reinforces the importance of recognizing KS as a high-risk thrombophilic condition and strongly supports lifelong anticoagulation following a first VTE event. Formal guideline inclusion is needed to standardize care for this underdiagnosed population. Additional research priorities include prospective risk-stratification tools, clarification of testosterone's net prothrombotic versus cardioprotective balance, and evaluation of whether thrombin generation testing can guide individualized anticoagulation decisions. Clinicians managing patients with KS should maintain a low threshold for thrombophilia workup, monitor hematocrit and relevant medication levels, including clozapine levels where applicable, and engage hematology for anticoagulation planning following any thrombotic event.
